# Pre-metastatic niche triggers SDF-1/CXCR4 axis and promotes organ colonisation by hepatocellular circulating tumour cells via downregulation of Prrx1

**DOI:** 10.1186/s13046-019-1475-6

**Published:** 2019-11-21

**Authors:** Yujun Tang, Yishi Lu, Yuan Chen, Lei Luo, Lei Cai, Bangjian Peng, Wenbin Huang, Hangyu Liao, Liang Zhao, Mingxin Pan

**Affiliations:** 1 Second Department of Hepatobiliary Surgery, Zhujiang Hospital, Southern Medical University, Guangzhou, China; 2 Department of Pathology, Nanfang Hospital, Southern Medical University, Guangzhou, China; 3 Department of Pathology, School of Basic Medical Sciences, Southern Medical University, Guangzhou, China; 4 Department of Hepatobiliary Surgery, the Fifth Affiliated Hospital, Southern Medical University, Guangzhou, China

**Keywords:** Circulating tumour cells, Neoplasm metastasis, Liver neoplasms

## Abstract

**Background:**

Circulating tumour cells (CTCs), especially mesenchymal CTCs, are important determinants of metastasis, which leads to most recurrence and mortality in hepatocellular carcinoma (HCC). However, little is known about the underlying mechanisms of CTC colonisation in pre-metastatic niches.

**Methods:**

Detection and classification of CTCs in patients were performed using the CanPatrol™ system. A lentiviral vector expressing Prrx1-targeting shRNA was constructed to generate a stable HCC cell line with low expression of Prrx1. The effect of Prrx1 knockdown on stemness, migration, and drug resistance of the cell line was assessed, including involvement of SDF-1/CXCR4 signalling. Promising clinical applications of an inhibitor of STAT3 tyrosine phosphorylation, C188–9, and specific blockade with CXCR4 antibody were explored.

**Results:**

The number of mesenchymal CTCs in blood was closely associated with tumour recurrence or metastasis. Pre-metastatic niche-derived SDF-1 could downregulate Prrx1, which induced the stemness, drug resistance, and increased expression of CXCR4 in HCC cells through the STAT3 pathway in vitro. In vivo*,* mice bearing tumours of Prrx1 low-expressing cells had significantly shorter survival. In xenograft tumours and clinical samples, loss of Prrx1 was negatively correlated with increased expression of CXCR4 in lung metastatic sites compared with that in the primary foci.

**Conclusions:**

These findings demonstrate that decreased expression of Prrx1 stimulates SDF-1/CXCR4 signalling and contributes to organ colonisation with blood CTCs in HCC. STAT3 inhibition and specific blockade of CXCR4 have clinical potential as therapeutics for eliminating organ metastasis in advanced HCC.

## Background

Hepatocellular carcinoma (HCC) is one of the most prevalent among human cancers that have high recurrence rates [1]. Hematogenous dissemination, which can lead to intrahepatic and distant metastases, is responsible for most cases of HCC recurrence [2]. Hematogenous metastasis is a complex process with many steps [3], and this process is closely correlated with the presence of circulating tumour cells (CTCs) in the vasculature [4]. In addition, because peripheral CTC detection is a simple, reproducible, and minimally invasive procedure, CTCs have been actively studied over the last few decades regarding their contributions to tumour recurrence and metastasis, as well as their utility in tumour diagnosis [5–7]. However, studies on the relationship between CTC subtypes and tumour recurrence/metastasis have rarely been reported.

Epithelial-mesenchymal transition (EMT), a reversible cellular program, leads to the detachment of epithelial cells from each other and the underlying basement membrane, and it converts epithelial cells into mesenchymal cell states [8, 9]. These mesenchymal cells have stem cell-like properties, increased motility and invasive capacity, resistance to several treatment strategies, and immunoevasive and immunosuppressive characteristics [10]. Our previous research has confirmed that the presence of mesenchymal CTCs (mCTCs) is an independent risk factor for the recurrence of HCC [11]. Although the transformation of epithelial-type tumour cells to a fully mesenchymal state rarely occurs during the progression of human cancers, we believe that EMT occurs during HCC metastasis, converting primary tumour cells to mCTCs. However, little is currently known regarding the underlying mechanisms of their contribution to HCC metastasis.

Stephen Paget proposed in 1889 that metastasis is dependent on the interaction between “seeds” (or cancer cells) and “soil” (the transfer microenvironment). A series of subsequent findings revealed that tumours induce the formation of microenvironments in distal organs that contribute to the survival and growth of tumour cells before they reach these sites [12]. These predetermined microenvironments are referred to as “pre-metastatic niches” (PMNs). Among the main substrates in these niches, stromal cell-derived factor-1 (SDF-1) is a critical chemokine that functions as a tumour metastasis promoter. C-X-C chemokine receptor type 4 (CXCR4)-expressing tumour cells migrate along the SDF-1 gradient to distant organs containing high levels of SDF-1 expression, eventually leading to metastasis [13]. Several studies have demonstrated that CXCR4 and SDF-1 play a critical role not only in guiding metastasis, but also in the development of liver cancer [14–16].

In the present study, we investigated the risk of recurrence in HCC patients with positive peripheral mCTCs. We further explored the mechanism of how the SDF-1/CXCR4 axis promotes organ colonisation by HCC CTCs.

## Methods

### Clinical samples collection

Thirty-six HCC patients (27 males and 9 females, from 20 to 73 years old, with a median age of 51.47 years), who underwent radical resection at Zhujiang Hospital of Southern Medical University from July 2015 to January 2017, were enrolled in this study. The inclusion criteria were as follows: (1) patients who underwent pathological specimen examination and had a definite pathological diagnosis of liver cancer according to the criteria set by the World Health Organisation; (2) patients who underwent radical resection by an experienced physician, with no residual lesions at the margins of the excision site as confirmed via postoperative pathology examination; (3) patients who had not been treated with other antitumour therapies before the resection; and (4) patients who had no extrahepatic metastasis confirmed by preoperative imaging. Tumour stage was determined according to the Barcelona Clinic Liver Cancer (BCLC) staging classification, and the degree of tumour differentiation was defined according to the Edmondson-Steiner grading system.

### Follow-up and tumour recurrence

The patients entered the clinical follow-up period to monitor for recurrence after their peripheral blood samples were collected postoperatively (3–35 days after the surgery, with a median of 18 days). The patients underwent various follow-up examinations and treatments after surgery according to routine clinical schedules. Recurrence was defined as intrahepatic recurrence and extrahepatic metastasis from a comprehensive diagnosis based on imaging results from computed tomography (CT), magnetic resonance imaging (MRI), and digital subtraction angiography (DSA), or from positron emission tomography (PET)-CT, serum alpha-fetoprotein (AFP) levels, and other examinations, with or without pathological diagnosis. Any evidence of recurrence was considered to be the end point. Time to recurrence (TTR) was defined as the time interval between resection and diagnosis of recurrence. Recurrence within 6 months after surgery was defined as early recurrence (ER).

### Detection and characterisation of CTCs using the CanPatrol™ system

We collected 5 ml of peripheral blood from 36 patients who were found to have space-occupying lesions in the liver via B-ultrasound diagnosis at Zhujiang Hospital affiliated with Southern Medical University between July 2015 and January 2017. We used CanPatrol™ CTC assay technology to isolate and count CTCs with different phenotypes in the patients’ peripheral blood. We designed three sets of nucleic acid probes to detect and characterize the expression levels of epithelial and mesenchymal genes in CTCs through multiplex RNA in situ hybridisation (RNA-ISH). This detection and characterisation of CTCs was as previously described [17]. The first set of probes contained four epithelial transcripts (CK8, 18, and 19; EpCAM), the second consisted of two mesenchymal transcripts (Vimentin and Twist) and the last contained only CD45 transcripts to distinguish between leukocytes and CTCs. Finally, the nuclei were stained with 4′, 6-diamidino-2- phenylindole (DAPI), and the cells were analysed using fluorescence microscopy. The red and green fluorescence signals observed in the cells represent the expression of epithelial and mesenchymal genes, respectively.

### Cell culture and treatment

The liver cell line LO2 and human HCC cell lines BEL-7404, Huh7, HepG2, and SMMC7721 were acquired from the Cell Bank of the Chinese Academy of Sciences (Shanghai, China) and maintained in Dulbecco’s Modified Eagle’s Medium (DMEM) containing 10% foetal bovine serum (FBS, Gibco, Carlsbad, CA, USA), 100 units/ml penicillin, and 100 mg/ml streptomycin sulfate. All cells were cultured in a humidified 5% CO_2_ incubator at 37 °C.

For inhibitor or cytokine treatment, both C188–9 (1 μg/ml in DMEM, Selleck Chemicals, Houston, TX, USA) and SDF-1 (Merck Millipore, MA, USA) were first dissolved in DMEM and then added to the cultured BEL-7404 cells at the desired concentration: 1 μM for C188–9 and 10 μM for SDF-1.

### Construction of paired related homeobox 1 (Prrx1) expression lentiviral vector

To generate PRRX1 low expression lentiviral vectors, we amplified the insert (full-length human PRRX1; NM_022716.2) by PCR from human reference cDNA. Lentiviruses were produced by transient transfection of 293 T cells with pSPAX2, pMD2G, and pHB-U6-MCS-zsgreen-puro (empty) (Hanbio, Shanghai, China) plasmid DNAs (2439 -BamHI and 2456 -EcoRI sites) plus LipoFiter™ (Hanbio Biotechnology, Shang Hai, China) following the manufacturer’s protocol.

### RNA isolation, reverse transcription, and quantitative PCR

Total RNA was extracted using TRIzol reagent according to the manufacturer’s protocol and our previous report [18]. To quantitate the expression of *Prrx1*, polyadenylated mRNA was purified from total RNA and subjected to reverse transcription. Quantitative PCR (qPCR) was carried out using SYBR® Green PCR master mix (Applied Biosystems; Foster City, CA) on an ABI 7500HT system. Glyceraldehyde-3-phosphate dehydrogenase (*GAPDH*) was chosen as the endogenous control. All primers were synthesised by Invitrogen (Shanghai, China). The expression level of each targeted gene was expressed as the fold change normalised to the control or reference group. Fold changes were calculated by relative quantification (2^-ΔΔCT^).

### Western blot and co-immunoprecipitation (co-IP) assays

Protein expression levels were assessed by immunoblot analysis of cell lysates (20–50 μg protein) in RIPA buffer containing 1× phosphate-buffered saline (PBS), 1% Nonidet P-40, 0.1% sodium dodecyl sulfate (SDS), 5 mM EDTA, 0.5% sodium deoxycholate, 1 mM sodium orthovanadate, and protease inhibitors. Primary antibodies to CD133 (11,000, Proteintech, CA, USA), SOX2 (1:1000, Proteintech), Nanog (1:1000, Proteintech), β-Actin (1500, Zsbio, Beijing, CHN), PRRX1 (11,000, Abcam, USA), STAT3 (11,000, Affinity, UK), p-STAT3 (11,000, Affinity), and CXCR4 (11,000, Proteintech) were used.

For co-IP assays, cells were transiently or stably transfected with the indicated constructs. The cells were harvested and lysed in 1 ml of lysis buffer (50 mM HEPES pH 7.5, 150 mM NaCl, 1 mM EDTA, 0.5% Nonidet P-40). The resulting lysates were subjected to immunoprecipitation with antibodies directed to the epitope tag. Immunoprecipitates were washed in lysis buffer, resolved by SDS-polyacrylamide gel electrophoresis, and subsequently analysed by protein immunoblotting.

### Transwell® migration assay

Cells in serum-free medium (1 × 10^5^ cells/100 μl) were added to the top chamber of each 8-mm-pore Transwell® chamber (Corning Star; Cambridge, Mass, USA). The bottom chamber was prepared using 10% FBS as a chemoattractant. The cells were allowed to migrate through the porous membrane for 24–48 h at 37 °C. Five randomly selected fields of cells were then counted under a microscope (original magnification, × 200). At least 4 chambers from 3 different experiments were analysed.

### Sphere formation assay

Cells were transduced with Prrx1 lentivirus or control lentivirus. At 24 h post-transfection, the cells were collected and seeded in 24-well ultra-low attachment plates in serum-free medium supplemented with 5 μg/ml insulin, 0.4% bovine serum albumin, 10 ng/ml basic fibroblast growth factor, and 20 ng/ml recombinant human epidermal growth factor for 2, 4, and 6 days. The size and number of spheroids were analysed under a light microscope (Olympus). The criterion for sphere formation was a spheroid larger than 50 μm in size. The data shown are the mean values of three independent experiments.

### Immunofluorescence (IF)

Cells were cultured on coverslips overnight, fixed with 4% paraformaldehyde for 20 min, and treated with 0.25% Triton X-100 for 10 min. After blocking in 10% normal blocking serum at room temperature for 10 min, the slides were incubated with antibodies at 4 °C overnight followed by washing with PBS three times. The coverslips were then incubated with fluorescein isothiocyanate (FITC)-conjugated or Texas Red (TR)-conjugated antibodies (1120, Santa Cruz) for 30 min at room temperature, and then stained with 4′,6-diamidino-2-phenylindole (DAPI; Invitrogen). The cells were observed under an Olympus FluoView™ FV1000 confocal microscope (Olympus, Hamburg, Germany). The images were acquired and analysed using OLYMPUS FLUOVIEW Ver. 4.2a Viewer software.

### Animals

All animal experiments were carried out with the approval of the Southern Medical University Animal Care and Use Committee in accordance with the guidelines for the ethical treatment of animals. Nude nu/nu mice were maintained in a barrier facility in racks filtered with a high-efficiency particulate air filter. The animals were fed an autoclaved laboratory rodent diet. The mice in this study were purchased from the Experimental Animal Centre of Southern Medical University, which is certified by the Guangdong Provincial Bureau of Science.

### Immunohistochemistry (IHC)

IHC was used to detect the expression of proteins in 3 μm sections from formalin-fixed, paraffin-embedded tissue specimens as described previously [19]. The slides were incubated overnight with primary antibodies against PRRX1 (1:200) and CXCR4 (1,200) at 4 °C. Mayer’s haematoxylin was applied for nuclear counterstaining. No antigen retrieval was performed. Positive controls and nonimmune protein-negative controls were used for each section. These sections were reviewed by three blinded pathologists, and the intensity of staining of malignant cells was scored to analyse the levels of protein expression as follows: 0 (no staining), 1 (weak staining, faint yellow), 2 (moderate staining, light brown), and 3 (strong staining, brown). Any discrepancies (overall < 5%) were settled by simultaneous re-evaluation.

### Statistical analysis

Data were analysed using SPSS statistical software version 20.0 (SPSS; Chicago, IL). Student’s *t*-test and one-way ANOVA were carried out for RT-qPCR. Significance of correlation between the expression of Prrx1 and CTC subtypes was determined using Pearson’s chi-squared (χ2) test. The correlation between PRRX1 and CXCR4 was determined using Spearman’s rank-order correlation. Kaplan–Meier plots were used to estimate the prognostic relevance of Prrx1 in univariate analysis. Statistical significance was established at *P* < 0.05.

## Results

### Number of mCTCs is associated with recurrence of HCC

**Fig. 1:**
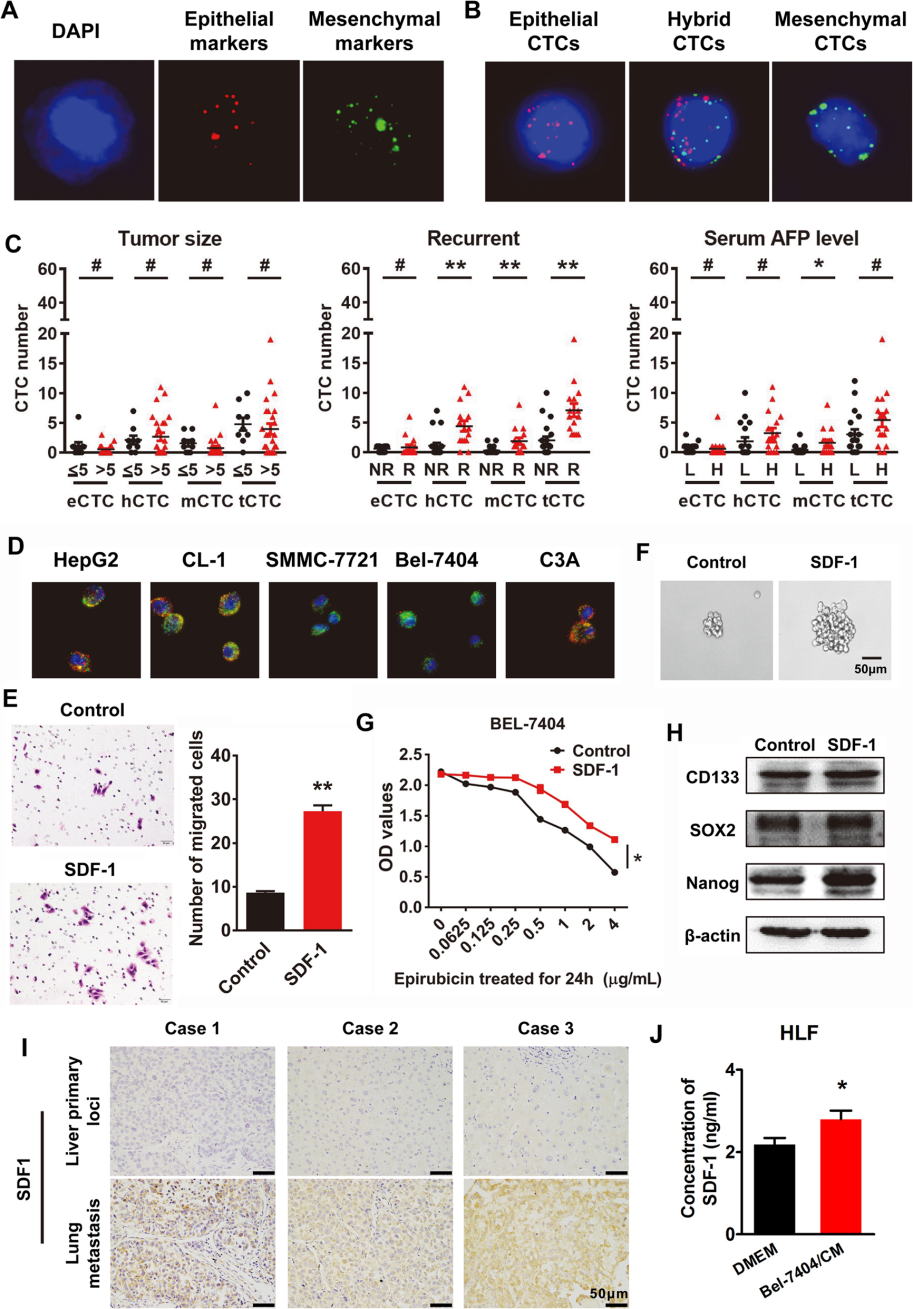
SDF-1 treatment induces stemness and migratory potential of HCC cells. **a** Nuclei were stained with 4′,6-diamidino-2-phenylindole (DAPI). The first set of probes contained four epithelial transcripts (CK8, CK18, CK19, and EpCAM), and the second set of probes consisted of two mesenchymal transcripts (Vimentin and Twist). The cells were analysed using fluorescence microscopy. The red and green fluorescent signals observed in the cells represent expression of epithelial and mesenchymal genes, respectively. **b** CTCs were classified as epithelial, hybrid, or mesenchymal CTCs according to the positive signals. **c** Numbers of various CTCs were calculated and their relevance to clinical parameters assessed. **d** Epithelial/mesenchymal phenotype was identified in 5 human HCC cell lines using the CanPatrol™ system. **e** Migrating cells were counted under a microscope in five randomly selected fields. Bars represent number of invaded cells. **f** Effect of SDF-1 on drug resistance (epirubicin) was evaluated by CCK-8 assay. **g** Phase-contrast images of sphere-forming assays of cells treated with SDF-1. **h** Western blotting was used to detect the expression of stemness markers. **i** Immunohistochemistry assay were performed to detected the expression of SDF-1 in primary HCC and lung metastastic foci. **j** ELISA assay were used to detect the concentration of SDF-1 in supernatant of indicated cells. # *P* > 0.05. * *P* < 0.05. * *P* < 0.01.

CTCs from 36 patients were identified using the CanPatrol™ system and followed up after surgery. These patients included 15 with recurrence (aged 26–73 years old with a median age of 50.8 years) and 21 without recurrence (aged 20–71 years old with a median age of 52 years). In IF assays, epithelial CTCs (eCTCs) and mCTCs showed red and green fluorescence, respectively. A hybrid population of CTCs expressing both epithelial- and mesenchymal-specific genes was also observed (Fig. 1a& b). As shown in Fig. 1c, more total CTCs, mCTCs, and hybrid CTCs were detected in the peripheral blood of the recurrence group than in the non-recurrence group after HCC radical resection. No difference was observed in the eCTC counts between the two groups. Moreover, the number of mCTCs was higher in patients with high AFP levels than in those with low AFP levels (Fig. 1c).

### SDF-1 treatment induces stemness and migratory potential of HCC cells

We identified the epithelial/mesenchymal phenotype in 5 human HCC cell lines using the CanPatrol™ system (Fig. 1d). The BEL-7404 cell line, which mainly expressed mesenchymal markers, was selected to simulate mCTCs in vitro. After treatment with SDF-1 (1 μg/ml, Peprotech, USA), a series of experiments were performed to analyse the biological characteristics of the HCC cells. As shown in the results in Fig. 1e–h, SDF-1 treatment could enhance migration (Fig. 1e), sphere formation (Fig. 1f), and resistance to epirubicin (Fig. 1g) of the cells, which was accompanied by increased expression of the stem cell markers CD133, SOX2, and Nanog (Fig. 1h). Immunohistochemistry assay revealed lower or no SDF-1 expression in primary HCC cells compared with that in lung metastatic foci (Fig. 1i). After treatment with conditional media of Bel-7404, increased secretion of SDF-1 was detected in the supernatant of human lung fibroblast (HLF) cells (Fig. 1j).

### Prrx1, as a downstream factor of SDF-1, contributes to lung colonisation of HCC cells

**Fig. 2:**
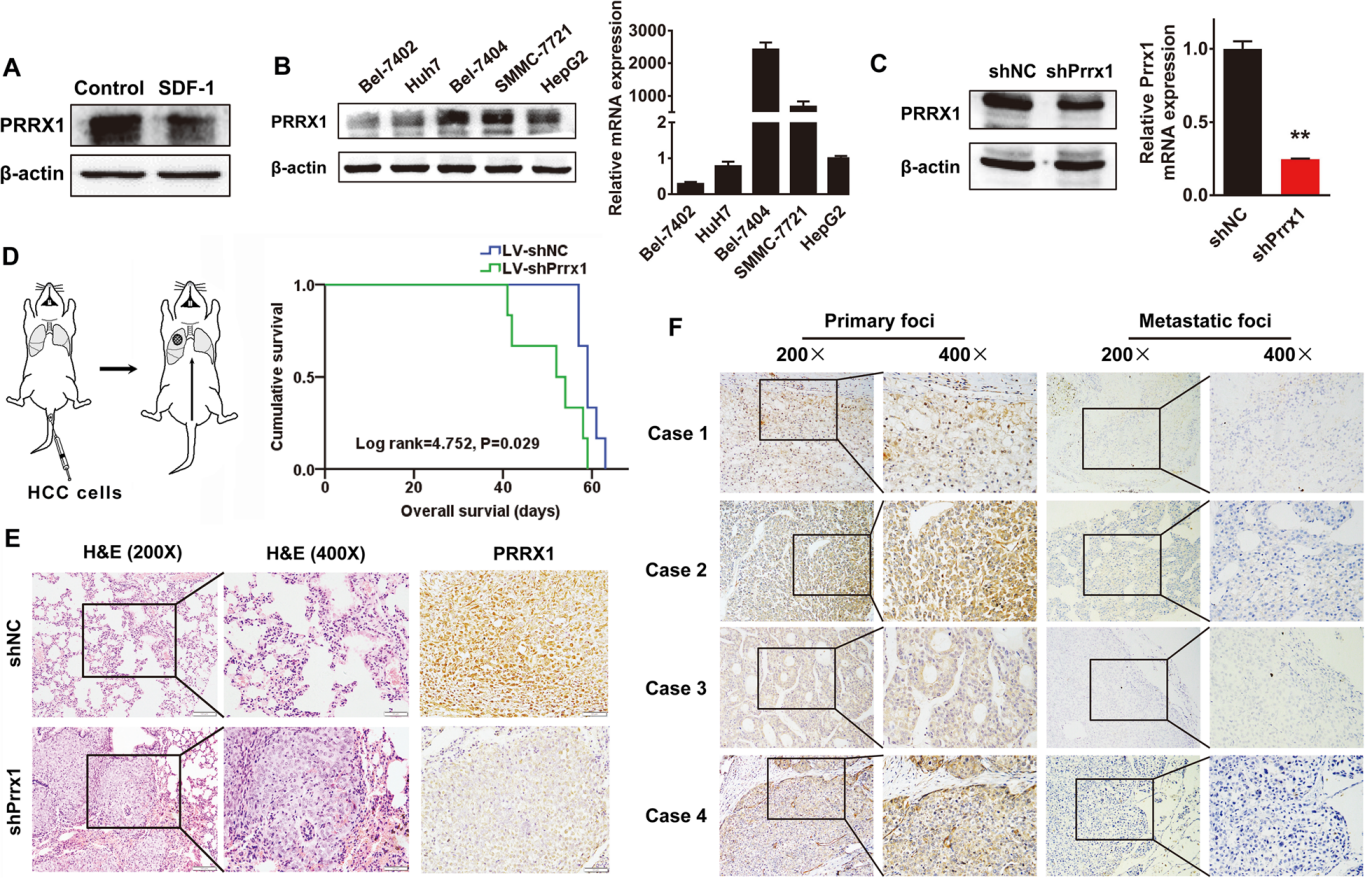
Prrx1, as a downstream factor of SDF-1, contributes to lung colonisation of HCC cells. **a** Western blotting of Prrx1 expression in SDF-1-treated cultured cells and control cells. **b** Protein and mRNA expression of Prrx1 in 5 HCC cell lines detected by western blot and RT-qPCR, respectively. **c** Bel-7404 cells were transduced with Prrx1 shRNAs. Western blot analysis of PRRX1 in two groups is shown. The signal was quantified using densitometric scanning software, and the protein abundance was normalised to β-actin. **d** Nude mice xenografted with indicated cells. Kaplan-Meier survival curves and univariate analyses (log-rank) for the xenografted mouse model are shown. **e** Representative photographs of haematoxylin and eosin-stained and PRRX1-stained colonisation nodules of the lung are shown. **f** IHC of PRRX1 in primary foci and lung metastatic sites of HCC patients.

Western blots showed that SDF-1 induces downregulation of Prrx1 protein expression (Fig. 2a). We investigated the expression of Prrx1 in the five HCC cell lines and found relatively high expression of both mRNA and protein in Bel-7404 cells (Fig. 2b). We generated Bel-7404 cells stably expressing low Prrx1 levels by shRNA lentivirus transduction, as demonstrated using western blots and RT-qPCR (Fig. 2c). Nude mice were injected in the tail vein with Bel-7404/LV-shNC or shPrrx1 cells to simulate HCC CTCs. Kaplan-Meier analysis showed that the shPrrx1 group had poorer survival than the control group (Fig. 2d). Compared with that of the controls, more and larger tumour nodules were found in the lungs of the mice that received cells treated with shPrrx1 (*P* < 0.05, Fig. 2e). An IHC assay to assess metastatic HCC also showed expression of Prrx1 that was significantly lower in the lung metastatic foci than in the paired primary tumour (Fig. 2f).

### Loss of Prrx1 is essential for SDF-1-induced stemness and migratory potential of HCC cells

**Fig. 3:**
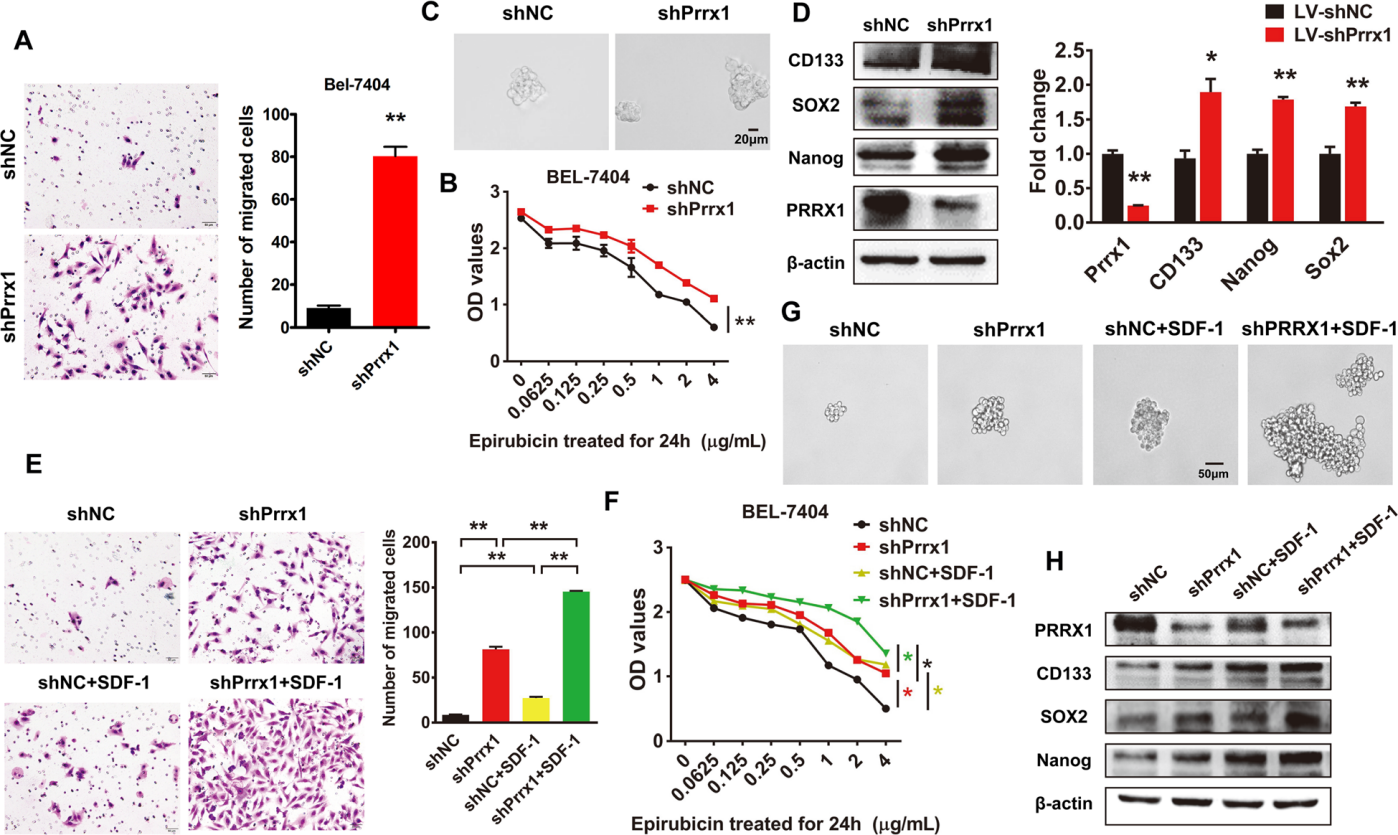
Loss of Prrx1 is essential for SDF-1-induced stemness and migratory potential of HCC cells. **a** Migrating cells were counted under a microscope in five randomly selected fields. Bars represent number of migrated cells. **b** Drug resistance (epirubicin) was evaluated in indicated cells by CCK-8 assay. **c** Phase-contrast images of sphere-forming assays of indicated cells. **d** Western blotting and RT-qPCR were used to detect protein and mRNA expression of stemness markers, respectively. **e** Invading cells were counted under a microscope in five randomly selected fields. Bars represent number of migrated cells. **f** Drug resistance (epirubicin) was evaluated in indicated cells by CCK-8 assay. **g** Phase-contrast images of sphere-forming assays of indicated cells. **h** Western blotting was performed to detect the expression of stemness markers. * *P* < 0.05, ** *P* < 0.01.

The Transwell® assay showed that knockdown of Prrx1 enhanced the migratory potential of Bel-7404 cells (Fig. 3a). Prrx1-silenced Bel-7404 cells were more capable of surviving after treatment with gradient concentrations of epirubicin than were the control cells (Fig. 3b). Further, downregulation of Prrx1 led to the formation of more and larger spheroids (Fig. 3c), accompanied by increased protein and mRNA expression of the stem cell markers CD133, SOX2, and Nanog (Fig. 3d). The rescue experiments showed that Prrx1 silencing could attenuate SDF-1-mediated cell migration (Fig. 3e), resistance to epirubicin (Fig. 3f), sphere formation (Fig. 3g), and expression of the stem cell markers (Fig. 3h).

### Knockdown of Prrx1 activates the SDF-1/CXCR4 axis via upregulating expression of CXCR4

**Fig. 4:**
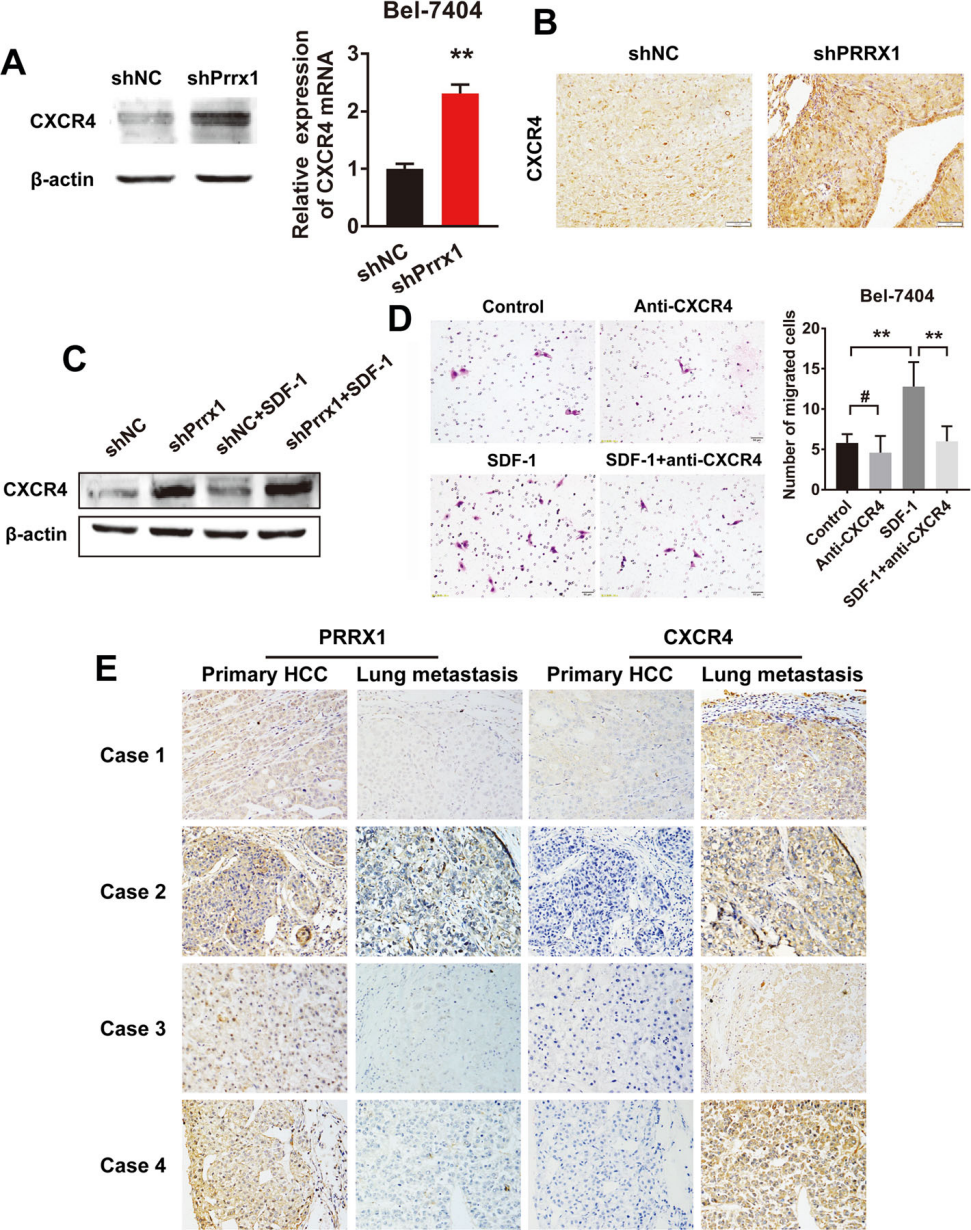
Knockdown of Prrx1 activates SDF-1/CXCR4 axis via upregulating the expression of CXCR4. **a** Western blotting and RT-qPCR were used to detect expression of CXCR4 in cells transduced with lentiviral shPrrx1. **b** IHC assay of CXCR4 in xenografted mouse lung metastatic sites. **c** Western blotting was performed to detect the expression of CXCR4 in cells co-treated with SDF-1 and shPrrx1. **d** Five randomly selected fields in the migration assay were counted under a microscope. Bars represent number of migrated cells. **e** IHC for CXCR4 was performed on serial sections of primary foci and lung metastatic sites of HCC patients. ** *P* < 0.01, # *P* > 0.05.

To explore the effect of Prrx1 on the surface expression of CXCR4, the specific receptor of SDF-1, we performed western blots and RT-qPCR assays in Prrx1-silenced cells. The results show that Prrx1 negatively regulates the protein and mRNA expression of CXCR4 (Fig. 4a). The IHC assay revealed that knockdown of Prrx1 increased the expression of CXCR4 lung metastatic nodules in xenografted mice in vivo (Fig. 4b). Prrx1 silencing could further increase the SDF-1-enhanced expression of CXCR4 (Fig. 4c). When CXCR4 antibody was used to block the interaction between SDF-1 and CXCR4, the stimulatory effect of SDF-1 on cell migration was significantly attenuated (Fig. 4d). Immunohistochemical examination of serial sections showed that, with decreased Prrx1 expression in lung metastatic foci, CXCR4 expression was significantly increased (Fig. 4e).

### Downregulation of Prrx1 induces the aggressive phenotypes of HCC cells via phosphorylation of STAT3

**Fig. 5:**
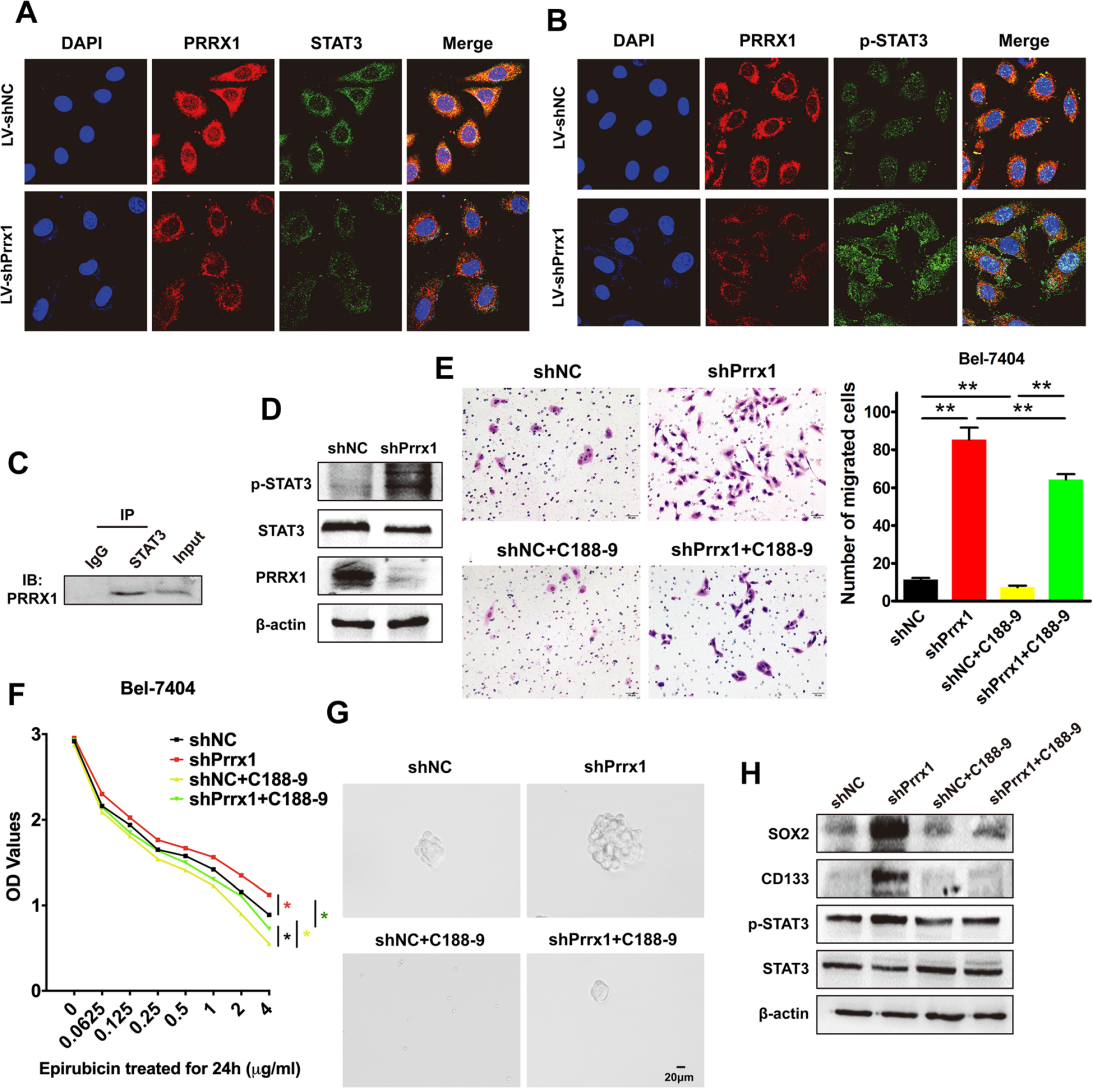
Downregulation of Prrx1 induces aggressive phenotypes of HCC cells via phosphorylation of STAT3. (**a** & **b**) Subcellular localisation of PRRX1 and STAT3 (**a**) or phosphorylated STAT3 (p-STAT3) (**b**) and their co-localisation in indicated cells was assessed by confocal microscopy (original magnification, × 2400). **c** Total lysates from Bel-7404 cells were subjected to immunoprecipitation with STAT3 antibody, followed by western blotting using PRRX1 antibody. **d** Western blotting was used to detect the expression of STAT3 and p-STAT3 in indicated cells. **e** Five randomly selected fields in the migration assay were counted under a microscope. Bars represent number of migrated cells. **f** Effect of STAT3 inhibitor, C188–9, on drug resistance (epirubicin) was evaluated in indicated cells by CCK-8 assay. **g** Phase-contrast images of sphere-forming assays of indicated cells treated with shPrrx11 and/or C188–9. **h** Western blot analysis of p-STAT3 and stemness markers in indicated cells. Protein abundance was normalised to β-actin. ** *P* < 0.01.

Prrx1 and STAT3 were co-localised, as shown in the IF assay (Fig. 5a&b). Prrx1 knockdown decreased the cytoplasmic expression of STAT3, whereas it enhanced the nuclear accumulation of phosphorylated STAT3. Prrx1 and STAT3 were shown to be associated by co-IP assays (Fig. 5c). Western blots revealed that decreased expression of Prrx1 induced the phosphorylation of STAT3 (Fig. 5d). A small molecule inhibitor of STAT3 tyrosine phosphorylation, C188–9 [20], could significantly attenuate Prrx1 knockdown-mediated cell migration (Fig. 5e), resistance to epirubicin (Fig. 5f), sphere formation (Fig. 5g), and expression of stem cell markers (Fig. 5h).

## Discussion

PMNs are the result of a combined systemic effect of tumour secretory factors and tumour exfoliated extracellular vesicles. Vascular leakage occurs first, followed by local stromal cell changes and recruitment of non-resident cells (such as bone marrow-derived cells) to these PMNs to attract CTCs. PMNs promote CTC survival and growth to promote organ transfer. Thus, in contrast to the metastatic niche that is initiated and shaped upon arrival of the CTCs, the PMN represents a microenvironment that is deficient in cancer cells, but abnormal and conducive to tumour growth [21].

**Fig. 6:**
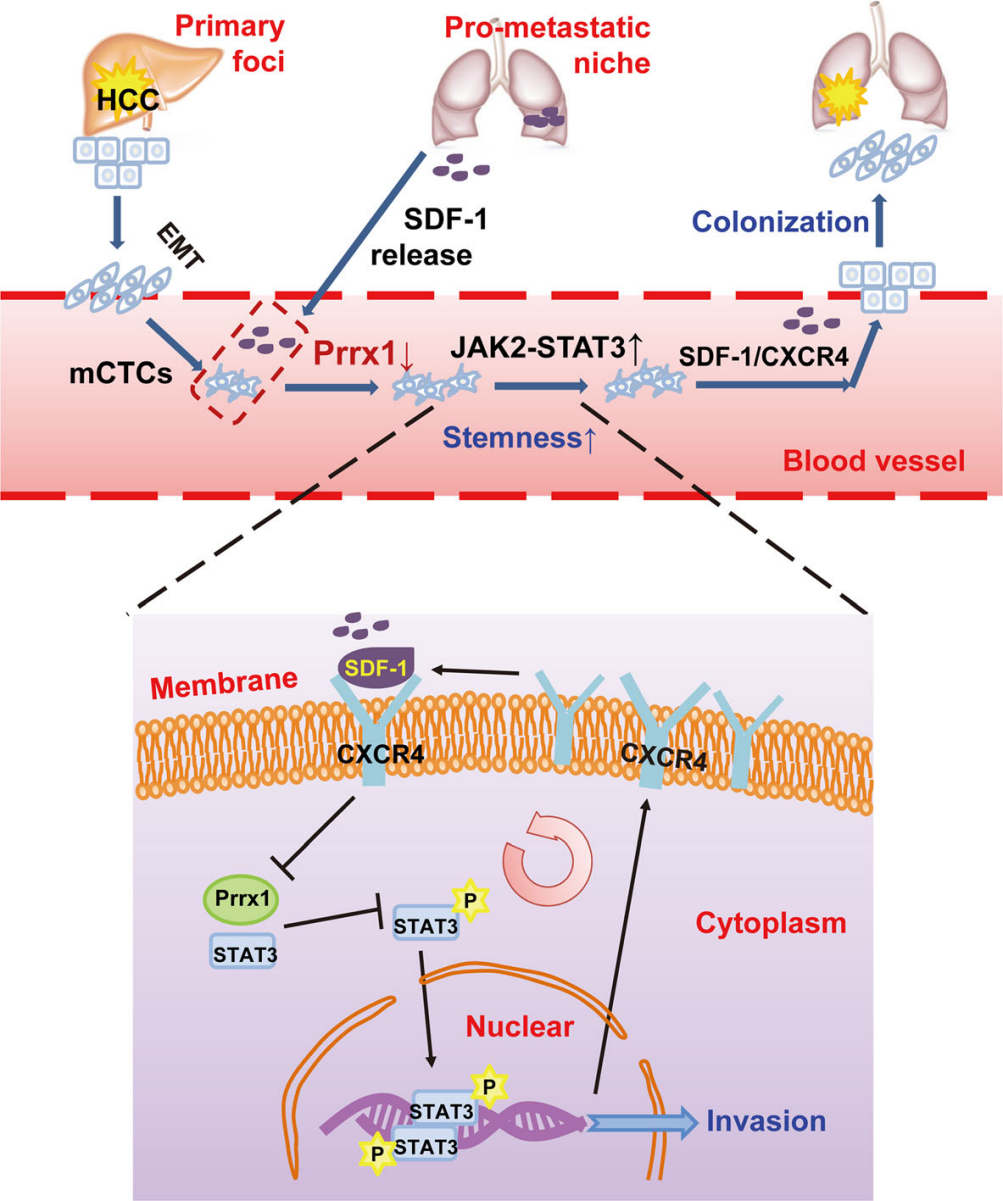
Model illustrating how pre-metastatic niches trigger SDF-1/CXCR4, promoting organ colonisation of CTCs via Prrx1 downregulatio

According to clinical and laboratory tests, the presence and number of mCTCs in the blood often reflects tumour recurrence and poor prognosis in patients with liver cancer. Furthermore, the SDF-1/CXCR4 axis promotes organ colonisation with mCTCs via downregulation of Prrx1. The deletion of Prrx1 upregulates the cell-surface expression of CXCR4 on mCTCs, which amplifies the chemotactic effect of SDF-1. SDF-1 in PMNs recruits CXCR4^high^PRRX1^low^ CTCs from the blood, finally contributing to the metastatic colonisation of distant organs (Fig. 6). Thus, it is known that the SDF-1/CXCR4 axis is involved in tumour metastasis, which plays an important role in the metastasis of digestive system tumours [14, 22, 23]. The present study demonstrates that deficiency of Prrx1 expression is critical for the role of the SDF-1/CXCR4 axis during tumour metastasis. Until now, the relationship between Prrx1 and HCC CTCs was unclear.

Previous studies have demonstrated that SDF-1 levels are significantly elevated in the peripheral blood of patients with advanced liver cancer with metastasis [24, 25]. At the same time, SDF-1 is widely expressed in various organs and tissues of the human body, but its expression is lower in normal liver or liver cancer tissues than in lung metastatic loci. TCGA analysis showed that a decreased expression of SDF-1 was found in HCC compared with normal liver tissues. Further, survival analysis showed that the expression of SDF-1 in the primary liver tumour is not associated with the prognosis of HCC patients with metastasis (Data not shown), indicating that the elevated SDF-1 in the peripheral blood of patients with advanced liver cancer is potentially derived from other organs. Moreover, over-activation of the TGF-β pathway promotes CXCR4 expression in HCC cells and confers CXCR4-dependent migration characteristics to cells to leave the primary tumour [26]. Our study has also demonstrated that HCC cells that have metastasised to the lungs have a high CXCR4 expression. Therefore, we consider that SDF-1 in the microenvironment induces the chemotaxis of circulating CXCR4-positive HCC CTCs to potential target organs.

Further, we believe that SDF-1 is derived from fibroblasts in the pre-metastatic niche. A number of studies have shown that SDF-1 expression in carcinoma-associated fibroblasts (CAF) promotes tumour cell growth, migration, and invasion [27, 28]. Exosome-derived miR-1247 of highly metastatic HCC cells induces CAF activation in the pre-metastatic niche of the lung, further promoting lung metastasis [11]. In addition, a recent research also demonstrated that the primary matrix stiffness in liver cancer leads to upregulation of LOXL2 secretion in HCC cells, which, in turn, promotes a series of changes in lung metastases and promotes the formation of pre-metastatic niche, including the promotion of secretion of SDF-1 from lung fibroblasts [29]. In our study, after treatment with conditional media of Bel-7404, the expression level of SDF-1 was increased in HLF cells. Therefore, we believe that during the process of HCC metastasis, elevated SDF-1 is mainly derived from fibroblasts in the pre-metastatic microenvironment of the lung.

Prrx1 was recently identified as an “inducer” involved in EMT. Prrx1 had to be downregulated to activate stem cell properties and allow colonisation [30]. Meanwhile, low Prrx1 expression is significantly associated with poor prognosis in various digestive system tumours, including colorectal cancer [31], gastric cancer [32], and HCC [33, 34]. In pancreatic cancer, Prrx1 has two alternatively spliced isoforms, Prrx1a and Prrx1b, which are similar in structure but have different, even completely opposite functions. Prrx1a promotes metastatic colonisation, tumour differentiation, and mesenchymal-epithelial transition, while Prrx1b promotes cancer cell invasion, tumour dedifferentiation, and EMT; each plays a role in different stages of pancreatic cancer development [35–37]. Herein, dynamic changes in Prrx1 expression were observed in the metastasis of blood CTCs to the lung. The mechanism underlying abnormal expression of Prrx1 in CTCs needs to be further explored.

The STAT3 signalling pathway has been reported to play an important role in the development and progression of HCC [38]. Activation of the STAT3 pathway (phosphorylation of STAT3) could promote stem cell properties and tumorigenicity in HCC [39]. Moreover, the STAT3 signalling pathway is closely related to the SDF-1/CXCR4 axis [40, 41]. CXCR4^+^ non-small cell lung cancer (NSCLC) cells are strong examples of tumorigenic stem-like cancer cells that maintain stemness through the CXCR4-mediated STAT3 pathway [42]. Now, we have also demonstrated that activation of the STAT3 pathway plays important roles in the survival, attraction, and colonisation of blood CTCs induced by SDF-1, which provides a potential therapeutic target for eliminating the organ metastasis of advanced HCC.

Due to the high genetic heterogeneity of liver cancer, traditional treatments often have great individual differences. Precise targeted therapy for liver cancer is now a developing trend. Considering its pleiotropic effects in tumour development, the CXCL12/CXCR4 axis is considered as a potential target for cancer treatment. Therefore, there have been many studies on the treatment of HCC with CXCR4 inhibitors [43–45]. Since SDF-1 can induce CXCR4-positive HCC cells to metastasize [26], there has also been a study to specifically reduce SDF-1 expression to inhibit HCC metastasis [46]. In our study, we focused on the effect of the CXCL12/CXCR4 axis on HCC CTCs, from a new perspective, to illustrate the significance of CXCR4 antibody in precision targeted therapy of liver cancer. However, the current research was conducted at the in vitro and in vivo levels. Therefore, more experimental and clinical data are needed to further explore potential therapeutic targets downstream and provide reliable evidence for targeted therapy of HCC.

## Conclusions

In summary, our findings suggest that the loss of Prrx1 expression is associated with the SDF-1/CXCR4 axis, which regulates the STAT3 pathway and synergistically influences HCC metastasis. In our study, use of a STAT3 signal pathway inhibitor or specific blockade with a CXCR4 antibody shows promising clinical applications. Further experiments are necessary to determine whether they have a positive therapeutic effect on HCC.

## Data Availability

Not applicable.

## Abbreviations

AFP: Alpha-fetoprotein
CTCs: Circulating tumour cells
CXCR4: C-X-C chemokine receptor type 4
DAPI: 4′,6-diamidino-2-phenylindole
DMSO: Dimethyl sulfoxide
EMT: Epithelial-mesenchymal transition
FBS: Foetal bovine serum
HCC: Hepatocellular carcinoma
IF: Immunofluorescence
IHC: Immunohistochemistry
mCTCs: mesenchymal circulating tumour cells
PBS: Phosphate-buffered saline
PMNs: Pre-metastatic niches
SDF-1: Stromal cell-derived factor 1
