# Correction to: Pre-metastatic niche triggers SDF-1/CXCR4 axis and promotes organ colonisation by hepatocellular circulating tumour cells via downregulation of Prrx1

**DOI:** 10.1186/s13046-020-01636-5

**Published:** 2020-07-07

**Authors:** Yujun Tang, Yishi Lu, Yuan Cheng, Lei Luo, Lei Cai, Bangjian Peng, Wenbin Huang, Hangyu Liao, Liang Zhao, Mingxin Pan

**Affiliations:** 1 Second Department of Hepatobiliary Surgery, Zhujiang Hospital, Southern Medical University, Guangzhou, China; 2 Department of Pathology, Nanfang Hospital, Southern Medical University, Guangzhou, China; 3 Department of Pathology, School of Basic Medical Sciences, Southern Medical University, Guangzhou, China; 4 Department of Hepatobiliary Surgery, the Fifth Affiliated Hospital, Southern Medical University, Guangzhou, China

**Correction to: J Exp Clin Cancer Res (2019) 38: 473**

**https://doi.org/10.1186/s13046-019-1475-6**

Following publication of the original article [1], the authors identified an error in the author name of Yuan Cheng.

The incorrect author name is: Yuan Chen

The correct author name is: Yuan Cheng

The author group has been updated above.
