# Correction to: Pre-metastatic niche triggers SDF-1/CXCR4 axis and promotes organ colonisation by hepatocellular circulating tumour cells via downregulation of Prrx1

**DOI:** 10.1186/s13046-021-02021-6

**Published:** 2021-07-16

**Authors:** Yujun Tang, Yishi Lu, Yuan Chen, Lei Luo, Lei Cai, Bangjian Peng, Wenbin Huang, Hangyu Liao, Liang Zhao, Mingxin Pan

**Affiliations:** 1 Second Department of Hepatobiliary Surgery, Zhujiang Hospital, Southern Medical University, Guangzhou, China; 2 Department of Pathology, Nanfang Hospital, Southern Medical University, Guangzhou, China; 3 Department of Pathology, School of Basic Medical Sciences, Southern Medical University, Guangzhou, China; 4 Department of Hepatobiliary Surgery, the Fifth Affiliated Hospital, Southern Medical University, Guangzhou, China

**Correction to: J Exp Clin Cancer Res 38, 473 (2019)**

**https://doi.org/10.1186/s13046-019-1475-6**

Following publication of the original article [1], the authors identified minor errors in image-typesetting in Fig. 3, specifically:

- Figure 3A: shNC image replaced with correct image
- Figure 3D: Nanog western blot replaced with correct image

The corrected figure is provided here. The corrections do not have any effect on the results or conclusions of the paper. The original article has been corrected.

**Fig. 3:**
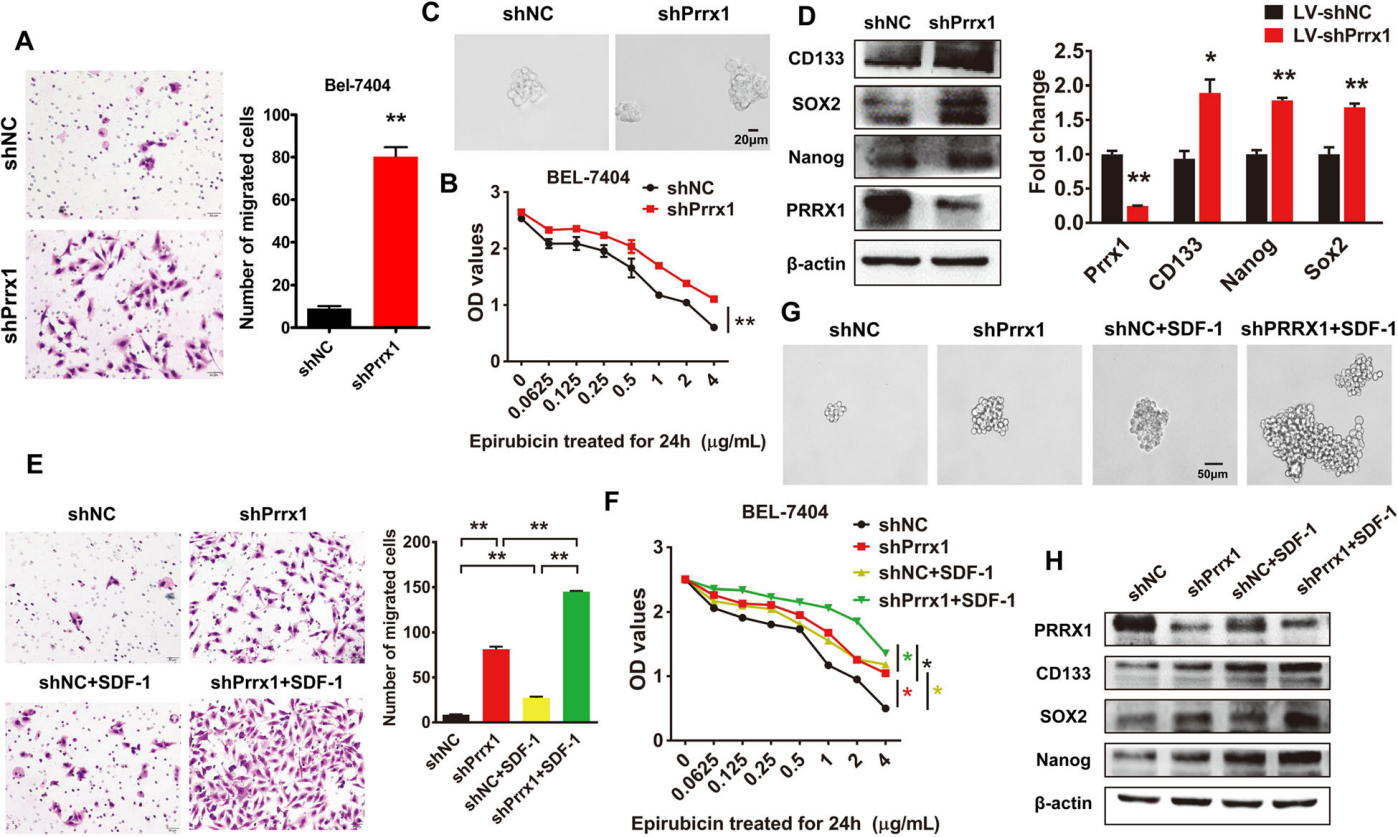
Loss of Prrx1 is essential for SDF-1-induced stemness and migratory potential of HCC cells. **a** Migrating cells were counted under a microscope in five randomly selected fields. Bars represent number of migrated cells. **b** Drug resistance (epirubicin) was evaluated in indicated cells by CCK-8 assay. **c** Phase-contrast images of sphere-forming assays of indicated cells. **d** Western blotting and RT-qPCR were used to detect protein and mRNA expression of stemness markers, respectively. **e** Invading cells were counted under a microscope in five randomly selected fields. Bars represent number of migrated cells. **f** Drug resistance (epirubicin) was evaluated in indicated cells by CCK-8 assay. **g** Phase-contrast images of sphere-forming assays of indicated cells. **h** Western blotting was performed to detect the expression of stemness markers. * *P* < 0.05, ** *P* < 0.01
